# McLean OCD Institute for Children and Adolescents: Overview, Rationale, and Description of Symptomatology and Functional Impairment

**DOI:** 10.3390/children12040505

**Published:** 2025-04-15

**Authors:** Alyssa L. Faro, Rebecca A. Wolenski, Chun W. Lee, Perihan Esra Guvenek-Cokol, Daniel P. Dickstein, Maria G Fraire

**Affiliations:** 1 McLean Hospital, 115 Mill Street, Belmont, MA 02478, USA; rwolenski@mclean.harvard.edu (R.A.W.); clee92@mgb.org (C.W.L.); pguvenek-cokol@mgb.org (P.E.G.-C.); ddickstein@mclean.harvard.edu (D.P.D.); mfraire@mclean.harvard.edu (M.G.F.); 2 Harvard Medical School, 25 Shattuck Street, Boston, MA 02115, USA

**Keywords:** OCD, adolescents, residential treatment

## Abstract

Background/Objectives: Residential treatment represents an important level of care for adolescents with severe and/or treatment-refractory obsessive–compulsive disorder (OCD). Despite accumulating evidence supporting the treatment efficacy and cost-effectiveness of insurance-based intensive OCD treatment in residential settings, few data exist that characterize the population of adolescent patients utilizing this level of care. As a result, residential treatment may be poorly understood by patients, their families, and referring providers, which may delay appropriate treatment for adolescents with OCD. Here, we characterize the patient population at an intensive residential treatment center (RTC) and partial hospitalization program (PHP) for adolescents (*Mage* = 15.23) with a primary diagnosis of OCD. Methods: We examine quantitative data collected from 168 adolescents admitted to the McLean OCD Institute for Children and Adolescents for the treatment of primary OCD or a related disorder over a three-year period. We also conduct analyses on a subset of patients (*n* = 120) who participated in the Child and Adolescent Routine Evaluation (CARE) Initiative (McLean Child Division-Wide Measurement-Based Care Program) to further characterize this patient population with a lens toward additional comorbidities and factors impacting prognosis. Results: The current paper describes the severity of symptom presentation, comorbidities, psychotropic medication profiles, and disruption to personal and family functioning. Analyses also include the prevalence of OCD subtypes and co-occurrence among varied presentations. Conclusions: In addition to identifying common clinical presentations in an RTC/PHP, this paper further aims to detail best practices and clinical rationale guiding a specialty RTC/PHP to inform families, providers, and payors about the individuals that most benefit from this level of care.

## 1. Introduction

***She has lost the ability to function independently.**** She is unwilling/unable to fully participate in everyday required activities, such as school, even online, and physical social interactions. We go through hundreds of plastic bags/gloves a week, she does everything with them on her hands, including sleeping and now the showering.*—parent of 13-year-old girl

*I miss just being able to **live my life freely**.*—16-year-old girl

Obsessive–compulsive disorder (OCD) is an impairing psychiatric disorder, particularly for children and adolescents. In severe cases, and particularly without intervention, symptoms impact many, if not all, domains of life and cause a significant decrease in quality of life [1]. Pediatric OCD can be a chronic condition with impairing symptoms that persist into adulthood despite initial pharmacological and outpatient psychological treatments [2], and it is associated with increased risks of suicide and substance use, among other serious conditions [3,4]. Psychiatric comorbidity is highly common in OCD, with some studies finding lifetime rates as high as 90% for individuals with OCD meeting criteria for another disorder [5]. Moreover, a 2022 meta-analysis [6] found strong associations between OCD and comorbidity with depression, bipolar disorder, psychosis, and substance use disorders highlighting the disabling impact of OCD. In addition to the substantial symptom burden of those struggling with OCD, the disorder negatively impacts the health and functioning of family members [7]. The impairment and risk factors associated with pediatric OCD indicate a need for specialized, early, and intensive treatment, such as can be provided in a residential setting.

Despite the debilitating nature of OCD, the average latency from onset to treatment is around seven years [8]. Additionally, OCD has one of the highest durations of untreated illness in severe mental disorders [8,9,10]. While significant symptom reduction can be achieved for many in outpatient settings, substantial symptom burden remains in nearly 50% of children even with combined treatment approaches [11]. Those who become “treatment resistant”—defined by Geller and March [12] as OCD patients who have not responded to cognitive behavioral therapy (CBT) and at least two adequate trials of selective serotonin reuptake inhibitors (SSRIs) or one trial of a serotonin reuptake inhibitor (SRI) and clomipramine—are often left with limited options and ongoing disability. At this stage, the severity of OCD symptoms and impairments can match that of individuals utilizing an inpatient level of care. The residential level of care offers high intensity, full-day services in a specialized psychiatric setting without reaching the psychiatric acuity of inpatient hospitalization. In effect, residential, specialized OCD treatment can keep children in need of these particular services out of general inpatient units, saving financial resources and reducing risks for iatrogenic effects. However, the residential level of OCD care may be poorly understood by both families of individuals with OCD and some referring clinical providers. As outlined below, we hope to increase awareness for families, referring providers, and even insurance payors of what this type of specialized, intensive treatment can offer for youth and families in need.

## 2. A Brief Overview of Pediatric Obsessive–Compulsive Disorder

### 2.1. Obsessions and Compulsions

OCD is defined by the presence of the following two core diagnostic features: obsessions and compulsions. Obsessions are a separate construct from excessive worries, which symptomatically categorize anxiety disorders, in that they include intrusive and repetitive thoughts, images, and urges. These obsessions are often considered “ego-dystonic” meaning the thoughts, images, or impulses themselves are unwanted (e.g., misaligned with an individual’s actual values, desires, or goals) and, therefore, can be highly distressing. For example, a child may experience intrusive and repetitive thoughts or images about personally harming their family members (e.g., “I am going to kill my family”), which is distressing because they do not actually want to inflict that harm. This contrasts with a child with generalized anxiety disorder characterized by excessive fear across a range of topics. Compulsions include repetitive, ritualized behaviors that an individual feels a strong urge to perform or complete in an effort to reduce the anxiety or discomfort elicited by the intrusive obsessions. The child mentioned above may compulsively clean their hands to “neutralize” the intrusive thoughts of harming their parents that they experience. Compulsions can also include mental rituals (e.g., counting, playing a scenario repetitively, and reassuring oneself in a specific way). The presence of obsessions and compulsions is time-consuming (e.g., requiring an hour or more of the individual’s time), distressing, and functionally impairing across domains. The DSM-5 [13] includes more than one specifier that is relevant to youth with OCD, including *insight*, indicating whether or not the individual recognizes that their OCD-related beliefs are true, e.g., whether an individual truly believes that their parent will die if they do not wash their hands for 120 s versus having insight into the likelihood of this event, nevertheless feeling compelled to complete the ritual [14].

In addition to these diagnostic criteria, one transdiagnostic factor that has recently garnered attention for its role in the development and maintenance of OCD is the cognitive vulnerability to intolerance of uncertainty (IU). IU is a characteristic whereby an individual regards uncertainty as unacceptable, negative, wrong, or even unsafe and, thus, has difficulty tolerating its occurrence (see Carleton’s [15] review for an exploration of the evolution of definitions). In an attempt to gain certainty, individuals with OCD may respond to uncertainty (e.g., about whether an obsession-related scenario will occur or has occurred) with maladaptive cognitions, emotional reactions, or behavioral reactions, such as rituals, compulsions, or other unhelpful choices [16]. IU has not only been implicated in OCD but also as a transdiagnostic trait across many psychopathologies. IU is stable and trait-like, and it has been identified as a specific treatment target amenable to intervention that we use in our program [17,18].

### 2.2. Prevalence

According to a recent DSM-5 report, OCD affects about 0.5–3% of youth [13]. An epidemiological study in Denmark found OCD has a cohort prevalence of 0.84% in *N* => 1 million youth [19]. Moreover, data suggest that youths with a history of OCD experienced significant increases in the frequency and severity of OCD symptoms throughout the COVID-19 pandemic [20,21]. Therefore, it is conceivable that current rates of OCD and symptom severity may be higher in youth in recent years. Previous studies have suggested that the incidence of OCD is highest in preadolescence, but distinct OCD symptomatology can be evident as early as 4–5 years of age [22,23]. The prevalence and severity of impairments starting in childhood indicate the importance of early intervention, e.g., as a benefit of providing treatment for youth [24,25].

### 2.3. Evidence-Based Treatment of OCD

The current evidence-based treatments of OCD include CBT and pharmacotherapy, with combined treatment offering considerable efficacy [11]. The prevailing CBT treatment for OCD has been exposure and response prevention (ERP), developed by Edna Foa and Alan Goldstein [26]. ERP is a cognitive-behavioral therapy stemming from the fear-learning research of early behaviorists. Traditionally, the goal of ERP has been to gradually habituate to the feared stimulus, which is based on emotional-processing theory. ERP is guided by fear hierarchies—a ladder of successively more challenging exposures—structuring treatment so that clients experience fear reduction for each stimulus prior to approaching the next exposure (see Riggs and Foa’s chapter in [27] for a detailed treatment description).

Despite combined treatment, a substantial percentage of children (around 50%) do not achieve symptom remission (see McGuire [28] for a meta-analysis). As such, the results of clinical trials focusing on immediate and sustained treatment gains have highlighted a need to improve therapeutic outcomes. Research aiming to maximize the impact of ERP treatment re-examined the learning model associated with ERP. The inhibitory learning model posits that fear-based associations cannot be unlearned but can be inhibited by forming new associations. Inhibitory learning shifts the focus of ERP to tolerating distressing emotional experiences (e.g., anxiety, disgust, shame, guilt, and fear) rather than habituating or experiencing a reduction in distress. Intervention focuses on strengthening this new learning, augmenting exposure with strategies such as increasing surprise from the exposure, conducting exposures in multiple settings and with varying fear levels, and emphasizing tolerance of distressing emotions rather than experiencing minimal distress or proving that the worst-case scenario did not come to pass [29,30]. One component of inhibitory learning is expectancy violations, which highlight the differences in the frequency and intensity of aversive outcomes from what was anticipated. An expectancy violation such as “I felt disgusted and enjoyed a meal prepared by someone else” can be highlighted for patients to demonstrate that multiple outcomes can exist in tandem.

Acceptance and commitment therapy (ACT) is congruent with the philosophical tenets taught in ERP and inhibitory learning models, and it has been studied as a conjunctive and standalone treatment for OCD. ACT is an experiential, transdiagnostic, cognitive-behavioral, and evidence-based treatment developed with a focus on living a valued life without attempting to control unwanted thoughts and emotions [31]. ACT consists of six primary concepts that together promote psychological flexibility. Psychological flexibility is the balance of present-moment awareness, committed action, acceptance, defusion, self-as-context, and values orientation. Through increasing psychological flexibility, patients are able to change their relationship with obsessions and emotional distress such that obsessions do not limit their lives. ERP incorporating inhibitory learning concepts further encourages living a valued life alongside uncomfortable thoughts and emotions.

Research examining the exact learning mechanisms of ERP and efforts to identify a treatment consistently providing superior outcomes is still emerging. Furthermore, individuals seeking residential care for the treatment of OCD rarely present without multiple psychiatric comorbidities [32,33] and, thus, transdiagnostic treatments with shared theoretical orientations are crucial to providing holistic care.

## 3. Present Study

### 3.1. Study Aims

The aims of this paper are twofold. First, we describe our specialized residential and partial hospitalization treatment program (RTC/PHP) for children and adolescents experiencing severe OCD and related disorders, including theoretical orientation and treatment modalities. Second, we explore quantitative and qualitative data collected from patients treated within our program to characterize the patient population of this oft-misunderstood level of care, highlighting the multiple comorbidities and impairments these adolescents face. While OCD often may improve in outpatient levels of care, when it does not, RTC offers a combination of intensive treatment, medication management, and wraparound services that cannot be achieved in other settings. Treatment resistant OCD is unfortunately prevalent and difficult to address when symptoms become pervasive within a patient’s life. In sum, the overall purposes of this paper are to inform families, providers, and payors about the symptom burden present at this level of care and to help better identify patients who are suited for specialty RTC/PHP.

### 3.2. Program Overview

#### 3.2.1. Clinical Intervention

Clinical intervention at OCDI Jr. is rooted in CBT with the integration of acceptance and commitment therapy (ACT) as consistent with inhibitory-learning-based ERP. Many of our residents report having tried ERP based on emotional processing theory, with limited success. Through treatment, we aim to directly increase psychological flexibility, tolerance of uncertainty, and values-oriented committed action.

While the clinical focus of our program is on reducing the impairments caused by OCD symptoms, we utilize transdiagnostic treatments to address the full system of presenting symptoms, pulling upon evidence-based behavioral treatments such as ACT, dialectical behavior therapy (DBT), and CBT. To the extent possible, by addressing symptoms from a transdiagnostic approach, we are able to reduce symptoms in multiple domains, e.g., OCD and depressive symptoms, rather than delaying treatment and further reinforcing a cycle of decline.

The residential setting offers therapeutic intervention in the milieu along with individual and group therapy, psychopharmacological treatment, and coached exposures. Patients are engaged in clinical programming for approximately school day hours, with time for limited academic work in the afternoon if appropriate. Evenings are a mixture of self-directed ERP, recreation, and structured activities of daily living (ADLs). Throughout the day trained ERP coaches are available to help residents resist compulsions that may interfere with their ability to complete a range of activities. Patients needing a residential level of care for the treatment of primary OCD often struggle to complete ADLs, such as caring for their hygiene and grooming, eating, and transitioning between tasks and spaces. Residential care provides treatment throughout the day as impairments present, rather than solely during designated treatment blocks. Coaching is offered in real time as OCD symptoms occur, whether it be at mealtimes, while brushing teeth, or on community outings. Planned coached ERPs can target tasks that would be difficult to achieve in the office, such as resisting rituals while dressing, showering, or toileting. In the milieu, staff are able to plan with residents how to approach the designated exposure (e.g., taking a shower) and then offer coaching from outside the bathroom while a resident practices showering for a shortened duration, resisting repetitive behaviors or allowing themself to come into contact with contaminated surfaces during the ERP.

Another therapeutic component of residential treatment is the milieu itself. Many of the patients completing our program have been socially isolated because of the severity of their psychiatric symptoms. Often the milieu is the first place patients have met another peer with OCD, and, in addition to finding camaraderie in the milieu, they also practice social skills and gain encouragement and support from their peers. For many, sharing physical space with peers (eating, talking, and even breathing) is its own exposure and, thus, an opportunity to resist engaging in a range of rituals and avoidance strategies. The milieu serves as such a vital element of treatment that programming has evolved to include team-based ERP activities for residents to benefit from learning from each other and observing their peers as they face and overcome their own challenges.

In addition to the individual treatment of symptoms, the program also aims to address the impact on the family system and promote treatment gains following discharge through two primary-caregiver-focused interventions. First, all patients in the program and their caregivers participate in at least once-weekly family meetings, which aim to provide psychoeducation, reduce accommodation and reassurance behaviors within the family system, plan for a successful discharge from the program, and prepare for the maintenance phase of treatment. In addition to the family meetings, the program also offers a multi-family psychoeducation group that focuses on providing caregivers with dedicated time weekly to learn many of the core treatment principles their children are learning, connect with others, and provide support through the treatment process.

#### 3.2.2. Setting and Treatment

The OCD Institute for Children and Adolescents (OCDI Jr.) at McLean Hospital is located in Belmont, Massachusetts, and offers an insurance-based specialty residential treatment program for adolescents (12–18) with primary OCD and related anxiety disorders. OCDI Jr. re-opened in a new location with new leadership, revised programming, and revamped data collection in the summer of 2020 to provide intensive exposure and response prevention (ERP)-based treatment and medication management in a 24/7 therapeutic environment. The program is designed to offer a stepped level of care, allowing patients to move into residential from the partial level of care as indicated, and similarly, residents are given the opportunity to step down into partial hospitalization before discharge. The partial program coexists within the residential program, such that patients share clinical programming and the milieu, only differing by whether they remain in the program overnight. Adolescents in the PHP range greatly in their current severity of symptomatology, as the program aims to serve those who are preparing to take the step into residential or transition down from residential, as well as those from the community who are seeking an intensive burst of treatment.

### 3.3. Patient Population and Admissions

In the present study, we analyzed data collected from the patients (*Mage* = 15.23, *SD* = 1.68, 71.2% assigned female at birth) (see Table 1) of a specialized OCD treatment program for children ages 12–18. All quantitative data were collected at admission as part of weekly data collected for the duration of each patient’s admission to the residential and/or the partial hospitalization program (PHP). A subset of our patient sample (n = 120 of 168) also participated in the Child and Adolescent Routine Evaluation (CARE) Initiative, the McLean Child Division-wide measurement-based care program [34]. The subset of data from CARE was collected at admission and allowed for a closer look at some domains of functioning that will be explored further below. The qualitative data added to this paper were gathered from both parent report at time of application to the program and from patient questionnaires at admission.

### 3.4. Quantitative and Qualitative Data

The study was conducted in accordance with the Declaration of Helsinki, and the clinical data collected internally at OCDI Jr. and through the CARE study were approved by the Institutional Review Board of MassGeneral Brigham (2022P003368, 4 January 2023; 2021P002913, 1 December 2021). All patients and parents in the CARE study provided written informed consent prior to participating in the study. Patient data that were not collected through CARE were part of a retrospective medical review and, thus, no informed consent was collected.

#### 3.4.1. Measures

**Obsessive–compulsive symptoms.** The Children’s Yale–Brown Obsessive-Compulsive Scale—Child Report [35] (CY-BOCS-CR) is a self-report measure of obsessive–compulsive symptoms and severity. It includes a checklist of symptom presentation and a reliable and valid 10-item scale to measure the severity of obsessions and compulsions. The measure has good internal consistency (α = 0.87) and has adequate internal consistency for CY-BOCS-CR obsession and compulsion severity scales (α = 0.78 and 0.81). In the present study, internal consistency was good for the CY-BOCS-CR total score (α = 0.89) and adequate for the obsession and compulsion severity scales (α = 0.82 and 0.83).

**Global functional impairment.** The World Health Organization Disability Assessment System 2.0 [36] is a 36-item assessment of functioning and functional impairment developed by the World Health Organization. The WHODAS 2.0 can be scored using either the “simple” or “complex” method, with the latter being used to compare scores to population norms. The WHODAS 2.0 has demonstrated acceptable to very good internal consistency at the domain and summary levels (α = 0.94) and (α = 0.98). In the present study internal consistency was excellent for the WHODAS 2.0 child (α = 0.94) and parent (α = 0.95) responses.

**Depressive symptoms.** The Center of Epidemiological Studies—Depression [37] (CES-D) is a 20-item self-report measure with good reliability and validity that asks individuals to rate the frequency they experienced depressive symptoms over the past week. The measure offers clinical cut-offs to assess the severity of depressive symptoms in a clinical population. The CES-D has excellent reliability (α = 0.90). In the present study, the internal consistency was excellent for the CES-D (α = 0.90)

**Anxiety.** The Spence Children’s Anxiety Scale—Child Version [38] (SCAS-C) is a self-report measure that includes 44 items that aim to capture symptoms related to generalized anxiety, panic, and/or agoraphobia social phobias, separation anxiety, OCD, and fears of physical injuries. The SCAS-C is a reliable and valid scale with excellent internal consistency (α = 0.90) and the subscales are also acceptable (α = 0.80, panic–agoraphobia; α = 0.71, separation anxiety; α = 0.72, social phobia; α = 0.75, obsessive–compulsive; and α = 0.77, generalized anxiety). In the present study, the internal consistency was excellent for the SCAS-C (α = 0.92) and the subscales were acceptable (α = 0.84, panic–agoraphobia; α = 0.77, separation anxiety; α = 0.80, social phobia; α = 0.84, obsessive–compulsive; and α = 0.83, generalized anxiety).

**Disruptive and inattentive symptoms.** Conners–Wells Adolescent Self-Report Scale [39] (CASS) was used to measure three symptom clusters related to conduct problems, cognitive problems/inattention, and hyperactivity–impulsivity, as well as an overall ADHD Index. The measure includes 27 items. The CASS has demonstrated excellent internal reliability, ranging from α = 0.83 to α = 0.92. Similarly, data from the current sample demonstrated excellent internal reliability (α = 0.93).

**Uncertainty Tolerance.** The Intolerance of Uncertainty Scale—Child Report [40] (IUSC) consists of 27 items used for the continuous measure of a child’s self-reported ability to tolerate general uncertainty. This is not a measure of anxiety symptoms, rather the items ask questions specific to ambiguity and uncertainty, for example, “Not knowing what will happen in the future makes life hard”, “When I am not sure of something I can’t work very well”, and “I should be able to prepare for everything in advance”. Higher scores indicate greater intolerance of uncertainty with scores ranging from 27 to 135. The IUSC has reliability, validity, and excellent internal consistency (α = 0.92). In the present study, the internal consistency was excellent for the IUSC (α = 0.96).

**Parent-report questionnaires.** Parents provided information on demographic data, family and patient history of medical and psychiatric disorders, past medication and therapeutic interventions, and school accommodations and answered open-ended questions about the impact of OCD in their child’s life and their own. Patients were also able to provide some qualitative data on their experience during the weekly data collection.

**Family accommodation.** The Family Accommodation Scale—Patient Version [41] (FAS-PV) is a self-report measure of accommodation in the family, specific to OCD, within the past week. The measure includes both a checklist of the types of accommodations and 19 items assessing the frequency of the accommodation behaviors on a 5-point Likert scale. While it was initially developed for use in adults, it was employed in this study because of the clinical fit. The FAS-PV has been found to have good internal consistency (α = 0.88). In the present study, the internal consistency was excellent for the CES-D (α = 0.91).

**Familial functioning.** The Family Assessment Device [42] (FAD) is a reliable and valid 60-item assessment of six dimensions of the McMaster Model of Family Functioning (MMFF). These dimensions measure affective involvement, affective responsiveness, behavioral control, communication, problem-solving, roles, and general family functioning. Previously, the FAD has demonstrated adequate (0.71) to good (0.83) reliability [43,44]. In the present study, internal consistency was good for the child (α = 0.86) and fair (α = 0.54) for parents.

#### 3.4.2. Data Analysis

Internal OCDI Jr. Data and CARE study data were captured through REDCap, a secure data capture system. Quantitative data, including demographic and those from the measures listed above, were analyzed in SPSS 28 to calculate the means and standard deviations of target variables.

## 4. Results

### 4.1. Patient Demographics and Admissions

Demographic information from our sample is presented in Table 1 below, including biological sex, age, race/ethnicity, and primary language spoken in the home. Note that we present data for our RTC sample broadly, as well as the subsample of participants who participated in the CARE study.

Of the 168 patients in this study, 21 of 168 (12.5%) completed only the partial hospitalization program (PHP). A total of 147 patients were admitted to the residential program, and 36.7% of residents also utilized the PHP level of care during their admission. Patients in the residential program are admitted for an estimated length of stay of two to three months (*Mresidential* = 54.00 days; *Mpartial* = 44.86 days) in the context of severe OCD that impacts their ability to function at home, school, and in their social lives.

### 4.2. Pediatric OCD Is Functionally Impairing Across Domains

*We are afraid that she has completely **allowed her fear to take over her life, changing who she is, her abilities, and aspirations.***—parent of 16-year-old girl

*I miss being able to share my art with others, doing quiet activities like reading and listening to music without my thoughts interrupting, being social in groups of people, and driving without being afraid of hurting other people or myself or being followed.*—17-year-old girl

**Figure 1:**
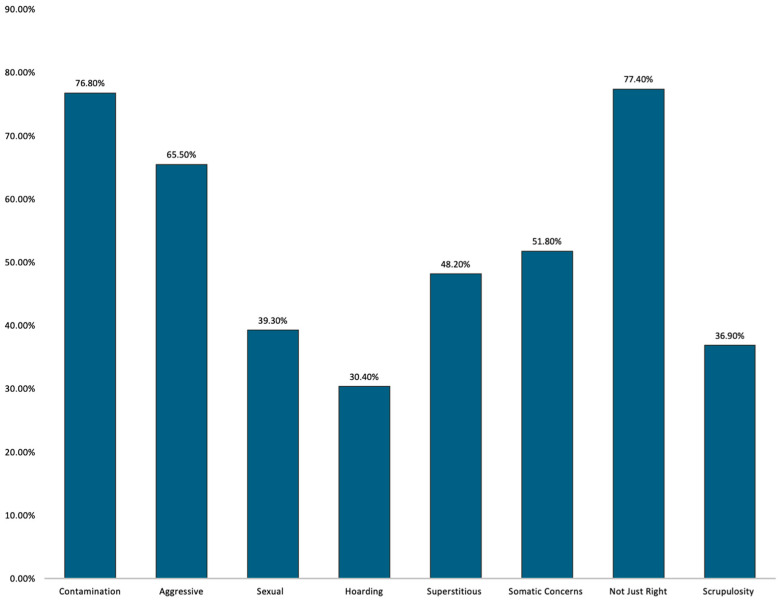
Frequency of OCD Subtypes at Admission.

Previous studies demonstrate that OCD not only negatively impacts youth on an emotional level (e.g., resulting in highly significant distress) but can often derail their functioning across multiple domains. In the current study, we found multiple domains of impairment across our patients’ lives. Patients presented to our program with severely impairing OCD at admission as measured by the CY-BOCS-CR (*M* = 25.06, *SD* = 7.06). In addition to presenting in the severe range, these patients frequently endorsed multiple OCD subtypes (*M* = 4.27, *SD* = 2.17), indicating the presenting complexity of these cases. Patients most frequently endorsed the “not just right experiences” (NJREs) subtype (77.4%) (see Figure 1), which has been associated with greater risk of relapse [45] (as well as more severe OCD symptom severity) [46].

The patients in our program and their caregivers reported highly elevated levels of global functional impairment as related to the impact of their OCD symptoms. Using the complex scoring method for the WHO Disability Assessment Schedule 2.0 (WHODAS 2.0), adolescents reported high levels of impairment (*M* = 34.25, *SD* = 18.5), which corresponds approximately to the 90th percentile compared to WHODAS 2.0 population norms. In other words, patients reported that in the context of their OCD, they have more challenges functioning across settings than 90% of the general population. Using the simple scoring method, patients reported an average WHODAS 2.0 score of *M* = 2.25 (SD = 0.69) and parents/caregivers reported an average score of *M* = 2.55 (SD = 0.72) at admission.

### 4.3. Comorbidities

*[Her] sense of self seems to be diminishing as she feels a loss of control because of OCD symptoms. She is showing signs of **depression**, and a new unwillingness to participate in the things she usually enjoys, because OCD interferes and takes away the pleasure.*—parent of 12-year-old girl

*Some things that I miss doing because **anxiety** or OCD got in the way is hanging out with friends without feeling anxiety, I miss playing the violin or singing without anxiety, and just really living my life the way I want to live it.*—15-year-old girl

Consistent with past research suggesting OCD in childhood is highly comorbid with mood, anxiety, and neurodevelopmental disorders [47], we found high rates of clinically significant symptoms of depression, anxiety, and ADHD.

#### 4.3.1. Depression

As measured by the CES-D, residents in our sample reported high levels of depressive symptoms (*M* = 32.62, *SD* = 11.24). The accepted clinical cutoff for the CES-D is ≥16 with higher scores indicating greater levels of depression.

#### 4.3.2. Anxiety Disorders

The SCAS-C was used to assess the presence of symptoms in six different anxiety disorders, including specific phobias, social anxiety, and panic disorder. T-scores above 60 represent the top 16th percentile and suggest clinically elevated anxiety. Overall, patients in our sample hovered in the clinically elevated range with overall anxiety scores (*M* = 60.75, *SD* = 9.10). Unsurprisingly, OCD symptoms were clinically elevated (*M* = 60.63, *SD* = 9.44) in the overall sample, with 28% of the patients reporting OCD symptoms in the 98th percentile. This pattern of elevated symptoms continued with **social anxiety** (*M* = 59.25, *SD* = 9.79); **panic/agoraphobia** (*M* = 60.68, *SD* = 9.15); **separation anxiety** (*M* = 59.90, *SD* = 9.88); and **generalized anxiety** (*M* = 61.57, *SD* = 9.05).

Notably, females reported higher symptoms in each subscale, reporting slightly higher **OCD** (*Mgirls* = 61.12, *SDgirls* = 9.77; *Mboys* = 59.38, *SDboys* = 8.50); **social anxiety** (*Mgirls* = 60.16, *SDgirls* = 9.05; *Mboys* = 56.89, *SDboys* = 11.24); **panic/agoraphobia** (*Mgirls* = 61.17, *SDgirls* = 9.16; *Mboys* = 59.40, *SDboys* = 9.08); **separation anxiety** (*Mgirls* = 60.02, *SDgirls* = 9.83; *Mboys* = 59.60, *SDboys* = 9.08); and **generalized anxiety** (*Mgirls* = 62.16, *SDgirls* = 8.95; *Mboys* = 60.07, *SDboys* = 9.25) symptoms than boys.

#### 4.3.3. ADHD

Various disruptive behaviors were measured with the CASS. With this scale, a T-score that falls between 41 and 59 is within the average range. A striking 87.7% of our patients’ hyperactivity subscale T-score fell above 90, which falls approximately above the 98th percentile [48]. A total of 23.6% of our sample’s cognitive problems/inattention T-scores fell above 90, with 71.7% scoring above 1 SD above the typical range. With regard to the overall ADHD index, 17.9% had a T-score above 90. Only 1.8% of our sample had a T-score in this index scale that fell within the average range.

#### 4.3.4. Intolerance of Uncertainty

Intolerance of uncertainty was measured by the IUSC, with an average score of 79.67 (*SD* = 25.66). Past findings in a pediatric IOP for anxiety and OCD found a mean score for the IUSC of *M* = 67.26 (*SD* = 24.09) [49]. Higher levels of intolerance of uncertainty have been associated with both higher levels of impairment and anxiety, as well as less reduction in functional impairment and anxiety following treatment [49].

### 4.4. Child/Individual Level Impairment

*We are now at a point where **OCD interferes with everything** that [he] needs to do. [He] does not get out of bed without help, he avoids basic hygiene routines like showering, brushing teeth, changing clothes. He does not make healthy food choices, does not participate in household chores or cleans up after his own messes… He seems to constantly be fighting intrusive thoughts, so he is often pacing around the house.*—parent of a 16-year-old boy

*I set barriers for myself that **make it difficult to do the things I normally do**, for example if I forgot to brush my hair, I would not do it for the rest of the week, and waste time thinking about if a week is the right amount of time to start over.*—12 year-old-girl

Interference in daily functioning was often reported in our patients’ “Activities of Daily Living (ADLs)”. For example, difficulty completing showers in a timely manner, brushing their teeth, an inability to feed oneself, dress oneself, get to sleep, or leave their rooms. Additionally, our data demonstrate that adolescents coming for treatment at the residential level of care endorse a heightened level of other interfering psychological symptoms, which can be a result of or exacerbated by OCD symptoms.

#### 4.4.1. School

*He is exhausted from his OCD anxiety at school and mostly sleeps or lays in bed after school and does not do homework. He had to **repeat 9th grade due to missed school** for therapy. He has failed first semester of 10th grade and is in danger of failing the rest of 10th grade.*—parent of a 17-year-old boy

***I don’t go to school anymore**** because of [OCD].*—15-year-old girl

Time-consuming rituals often greatly hinder youths’ ability to attend school regularly. Many of our patients have not consistently attended school for months or years prior to their admission, and, as a result, 10.2% reported having repeated a grade. Our patients commonly receive services from the school counselor or psychologist (57.8%), as well as support through Individualized Education Programs (IEPs) (38%) and 504 Plans (49.4%). Our adolescent sample reported a mean score of *M* = 47.32 (*SD* = 32.22) on the WHODAS 2.0 school subscale, indicating greater difficulty completing school responsibilities than approximately 94% of the general population. Despite this striking data about the academic struggles of our patient population, we also highlight that 10.2% of patients are or were enrolled in a gifted and talented program, which is substantially higher than the national rate of 6.6% [50].

#### 4.4.2. Social

***He’s not interacting with friends****. He’s not really interacting with his brother anymore.*—parent of a 13-year-old boy

*I miss being able to **go anywhere I wanted** without feeling contaminated and **being able to spend time with friends** without OCD and anxiety getting in the way.*—15-year-old girl

Our adolescent sample reported an average score of *M* = 34.42 (*SD* = 24.5) on the WHODAS 2.0 “getting along” subscale. This indicates that our patient sample tends to have more difficulty interacting with other people who they might be close to or less familiar with, than about 90% of the general population. OCD can impact an adolescent’s willingness to engage socially in numerous ways. For instance, in an effort to avoid triggering intrusive thoughts (e.g., harm obsessions, contamination obsessions, scrupulosity concerns, and many more), adolescents may choose to avoid interacting with other people altogether. Other times, obsessions (e.g., an excessive concern with saying the right or wrong thing) could negatively impact an adolescent’s ability to fluidly and typically communicate with others. Another common compulsion is reassurance-seeking (i.e., attempt to gain reassurance from others), which adolescents may engage in not only with their parents but also their peers. In line with other rituals and compulsions, the relief gained from receiving reassurance from others is typically only temporary, which makes the behavior repetitive and intrusive in an interaction.

### 4.5. Prior Treatment Trials

#### 4.5.1. Prior Psychosocial Treatment

Per parent report, before seeking care at the residential level, most (95.2%) patients had previously engaged in at least some form of psychological therapy. More than half (58.4%) had already received treatment at the intensive outpatient level (IOP), partial hospitalization, or psychiatric inpatient before admitting to our residential program, indicating multiple attempts at intervention with limited success.

#### 4.5.2. Psychiatric Medication Treatment

Parent-reported data at the time of program referral were available for 166 of the 168 patients (see Table 2). The majority (84.9%) of all patients in our sample were actively receiving an SRI at the time of referral. The most common SRI classes were SSRIs (75.9%) followed by serotonin–norepinephrine reuptake inhibitors (SNRIs) (6.6%) and clomipramine (4.2%). Despite the severity of the symptoms, 7.8% of patients were completely SRI-naive at the time of their referral.

Nearly one-quarter of our sample (24.1%) had two or more past trials of SRIs with incomplete responses. For patients with limited responses to SRIs and/or a persistently significant symptom burden despite SRI treatment, augmentation with low-dose second-generation antipsychotic medication (SGAs) is perhaps the most validated pharmacological strategy for treatment-refractory OCD, particularly those with comorbid tic disorders [51]. Other than SSRIs, SGAs were the most frequently reported psychiatric medication in our sample with more than one-third of patients actively receiving an SGA at the time of referral (37.3%). Risperidone and aripiprazole were the most frequent active SGAs (10.2% and 9.0%, respectively) and previously trialed SGAs (5.4% and 13.9%, respectively; see Table 2). This is consistent with evidence from adult trials and the few extant pediatric studies supporting the efficacy of risperidone and aripiprazole among SGAs in treatment-resistant OCD [51,52].

Polypharmacy was common with 51.8% of our sample concurrently taking ≥3 medications and 17.5% taking ≥5 medications at the time of referral. However, when the number of psychiatric medications was isolated by excluding non-psychiatric medications and supplements, these percentages decreased to 38.0% receiving ≥3 psychiatric medications and 4.2% receiving ≥5 psychiatric medications. A proportion of this medication burden is thought to reflect the medical complexity of our patient population. In addition, approximately one in five patients (21.7%) in the sample reported comorbidity with a chronic or active medical issue. Moreover, 44.6% of our sample population reported at least one allergy, which is in contrast with the 27.2% of youths in the community, based on data from the Centers for Disease Control and Prevention [53]. This greater burden of allergies in our sample is of potential interest as correlations between immune function and psychiatric diseases have been previously identified but remain poorly elucidated [54].

### 4.6. Family

#### 4.6.1. Family Psychiatric History

More than three-quarters (75.9%) of our sample had a reported family history of psychiatric disorders, with 46.4% of parents indicating a personal history of severe anxiety. Nearly one in four caregivers reported a suicide attempt within the family, including the patient, further underscoring the prevalence of mental illness within these families. This is particularly notable given recent attention to the greater risk of dying by suicide faced by those with OCD [3,55] and the contributing risk of a family history of suicide attempts (see Favril et al. [56] for a meta-analysis). The rate of family psychiatric illness highlights another stressor potentially contributing to the overall well-being of these adolescents, and the difficulties parents may face as they confront their own emotions about their child’s struggles.

#### 4.6.2. Family Impact

***Our house is run by his OCD**** … He needs constant reassurance and involves mom in most of the compulsions (saying things a certain way) to ease the intrusive thoughts.*—parent of 14-year-old boy

*I miss having fun and going out with my family, especially on the weekends. I miss enjoying the moment and not thinking ahead always. **I miss talking with my family** about topics that aren’t related to planning/are serious or are about my anxiety.*—12-year-old girl

Within our sample, the mean FAS score was *M* = 23.87 (*SD* = 16.36), indicating a similar level of familial accommodation found in an adult residential OCD program and was associated with OCD severity [57].

Within our sample, 120 adolescents and 64 parents completed the FAD. In both the child and parent samples, affective involvement was rated as the least healthy/highest dysfunctional area of family functioning (*Mchild* = 3.10, *Mparent* = 3.00), landing well above the clinical “unhealthy” cut-off of 2.1 [44]. Problem-solving was rated as the healthiest/least dysfunctional (*Mchild* = 2.14, *Mparent* = 2.05) in both samples. Our data suggest that children and parents were aligned in the assessment of familial functioning strengths and weaknesses. Affective involvement refers to the extent to which family members are involved in and interested in the emotions, behaviors, or interests of other family members and includes items such as, “Even though we mean well, we intrude too much into each other’s lives”. While we cannot say for certain what each patient and parent meant when completing this scale, one interpretation of this finding could point to how children with OCD are reinforced by parents becoming involved in their lives (e.g., accommodating and engaging in rituals for them), whereas parents identify the burden of becoming heavily involved.

While the idea of having their child attend a residential program may initially seem daunting to families, it offers a unique opportunity to target the family-involvement cycle by limiting parental accommodations, reassurance providing, and providing daily opportunities for youth to autonomously resist the urge to engage in compulsions.

## 5. Discussion

As the conversation about OCD has increased in recent decades, the disorder has been mischaracterized, misunderstood, and often downplayed in the public eye. Numerous myths about OCD are circulated, including that is it a quirk rather than an impairment, many are “just a little bit OCD”, and that there is little hope for those suffering [58]. Even within the mental healthcare arena, we find that providers may not always have the required training in evidence-based practices for the treatment of OCD. Additionally, this leaves families not only with less access to evidence-based care but also reduces the likelihood that they become familiar with the option for evidence-based residential care.

We hope to address these gaps in knowledge through this paper. We outlined both quantitative and qualitative findings collected directly from adolescents, and their caregivers, treated within a specialty OCD treatment program to clarify who utilizes this level of care. Growing evidence strongly suggests residential treatment is an efficacious option for adolescents with severe and/or treatment-refractory OCD. However, information characterizing the consumers of residential care is not readily available, such that patients, their families, and their providers may not be aware of this option or be able to assess when a patient would most benefit. We also described this specialty treatment program and the rationale for this treatment setting in hopes that this information can improve understanding for patients and providers and reduce delays of appropriate treatment for adolescents with OCD.

Overall, patients arrive at our treatment program with severe and complex OCD. In addition to their primary concern of OCD and associated functional impairments, patients at this specialty program presented with a variety of comorbid diagnoses (e.g., depression, anxiety disorders, and ADHD). While our patients are being treated for a primary OCD diagnosis, it is quite notable to juxtapose their degree of depressive symptomatology with adolescents in other levels of care. For example, when compared to adolescents in inpatient treatment, the degree of depressive symptoms is nearly equivalent with the mean score in our sample being just slightly higher [59]. Our data also strongly suggest that our sample struggles with clinically elevated levels of hyperactivity, cognitive challenges (inattention), and overall ADHD symptoms, which aligns with previous findings that ADHD is a common comorbidity observed in early-onset OCD [60].

Additionally, the negative impact of OCD symptoms was pervasive and significant in our patients’ lives. Adolescents and their caregivers described how OCD symptoms negatively impacted them personally, academically, socially, and in the context of their family system functioning. Most striking was the evidence, taken from both quantitative measures of impairment and qualitative descriptions, which suggests that the adolescents’ lives were significantly altered by the presence of OCD symptoms and their efforts to reduce the associated distress. Our patients reported they have more challenges functioning across settings than 90% of the general population. For comparison, we looked at previous child and adolescent WHODAS 2.0 reports around the world. In a sample of 188 adolescents receiving care in psychiatric hospitals in Mexico, the mean WHODAS 2.0 score was *M* = 2.17 [61] compared with our sample’s *M* = 2.25. In a sample of 92 Canadian child–parent dyads, of whom the adolescents were receiving inpatient or outpatient mental health care at a pediatric hospital, the parents’ mean WHODAS 2.0 score was *M* = 2.32 [62] compared with our sample’s *M* = 2.55.

Our patients and their families also reported significant familial distress. In line with our findings, previous studies demonstrate that parents and caregivers of youth with OCD experience heightened levels of frustration and helplessness [63]. Many family routines are impacted, including morning and bedtime routines, as well as mealtimes in the home [64]. Parental and sibling relationships with the individual and the family’s ability to engage in enjoyable activities together are often negatively impacted. For example, parents in our sample reported difficulty speaking with their children due to their child engaging in ritualized interactions (e.g., avoiding certain words, seeking reassurance, and speaking in a way that stilts the flow of conversation), avoiding family members altogether, or asking family members to engage in compulsions for them (e.g., repeat something until it sounds “just right”). Unfortunately, the burden of OCD can also be financial. Through our sample, multiple parents reported having to leave their employment to be available to support their child with OCD (i.e., due to the sheer time it takes to attend to a child’s symptoms), and spending money to buy items related to OCD symptoms (e.g., cleaning supplies, new clothes).

These comorbidities and functional impairments tend to exacerbate OCD symptoms, interfere with treatment success at a lower level of care by limiting the ability to meaningfully engage in exposure treatment, and require a more nuanced approach to care. Despite the majority of adolescents seeking some form of psychological or pharmacological intervention previously, including intensive levels of care such as IOP and inpatient admissions, a residential level of care was still required to target OCD symptoms. We take this data to suggest that other levels and avenues of care may not always be sufficient to reduce OCD’s impact when it is severe and other contributing factors are at play, such as comorbidities, high familial accommodation, and other stressors.

To further compound the burden our families experience, insurance companies are often less experienced with the debilitating nature of OCD and reluctant to cover higher levels of care, instead utilizing general inpatient criteria to determine whether treatment is medically necessary. Those who seek our treatment services are significantly impaired, often to the point they are no longer able to access outpatient services effectively, while simultaneously not meeting inpatient hospitalization criteria despite facing similar challenges with daily functioning. Despite previous treatment, multiple medication trials, additional school resources, and extreme family support, those who seek our services still require more care, particularly given their multiple risk factors for further decompensation, suicide risk, and complex comorbidities. Despite a similar degree of impairment to patients in an inpatient level of care, the residential level is not suited for those who are acutely psychotic, suicidal, or homicidal. Thus, the communication between insurance companies using inpatient criteria and providers of residential treatment can result in the denial of critical evidence-based residential services.

Fortunately, residential treatment provides intensive and individualized care for youth who need it most. Our data not only reflect the very real clinical complexity of youth struggling with OCD, but it also highlights the need for targeted evidence-based treatments to reduce symptoms and improve quality of life. To provide comprehensive care, our program is designed to support those with individual treatment plans, groups focused on exposures and skills building, family meetings and parent support, and in-the-moment exposure coaching. There is consistently high demand for our program, yet, as of this writing, only two OCD-specific, insurance-based residential treatment programs for adolescents exist in the United States.

Our findings also provide further support to the clinical design of our treatment. While the current evidence-based treatments of OCD emphasize the combination of CBT and pharmacotherapy, almost 50% are not considered treatment responders [11,28]. Within our sample, 95% of our patients had previous therapy experience and 58% had received higher level of care treatment. While our treatment is still rooted in the foundations of focusing on exposure and response prevention and pharmacotherapy, at the individual level, we intentionally emphasize the inhibitory learning process of ERP, values-based decision making, and the skill of tolerating uncertainty. Our sample reflected a high degree of difficulty with tolerating uncertainty, which is a transdiagnostic factor that has garnered recent attention both in the treatment of anxiety and OCD [65,66], as well as eating disorders [67] and the prevention of suicidality [68]. In our program, we intentionally evaluate and target the ability to tolerate uncertainty because a prominent component of OCD is seeking certainty through rituals or reassurances. We aim to help our patients learn to tolerate uncertainty by providing psychoeducation around the concept, language to help them better communicate their experience, and the skills to sit with the discomfort of uncertainty.

As evidenced by the qualitative and quantitative data from our sample, OCD is an incredibly impairing disorder for young people, and it affects more than the individual. The family system most often becomes heavily enmeshed in the demands and challenges of the disorder. This frequently manifests as caregivers constantly accommodating OCD-related rituals, providing reassurance, and accommodating the child in avoiding people, places, or situations that may trigger intrusive thoughts or lead to time-consuming compulsions. Often, caregivers accommodate their child’s OCD in an attempt to alleviate the child’s anxiety, which parallels the function of compulsions and rituals by providing *temporary* relief to the child while also reinforcing the relationship between the behavior and the obsession. These accommodations inadvertently maintain the cycle of OCD and further entrench the family system in the cycle of obsessions–distress–compulsions–temporary relief. As such, our program provides weekly family meetings to discuss the OCD cycle and ways it is inadvertently reinforced, how to reduce accommodations, and ways to support the individual while not supporting their OCD. Additionally, we provide a weekly parent group that discusses treatment components, as well as provides parents a platform for additional support.

### 5.1. Future Directions

Moving forward we aim to better understand how this pattern of multiple comorbidities, multiple OCD themes, and high functional impairment may predict and influence treatment outcomes. Additionally, we will seek to further elucidate specific transdiagnostic processes that facilitate a reduction in OCD and anxiety symptoms. Given the complex nature of our population, we have the opportunity to better understand how evidence-based treatments work in an intensive residential clinical setting.

One of the aims of this project is to move the needle in terms of a better understanding of who seeks the services of a specialty residential program and why it is needed. Our hope for the future is that those seeking services learn about specialty residential options earlier in their treatment journey and insurance companies recognize the value of covering these services.

### 5.2. Limitations

Our study is limited by design in that we are specifically focusing on one timepoint in our sample in order to better characterize those who seek our services. Our sample is restricted to those who meet our admission criteria, which includes a primary OCD/anxiety disorder and a willingness to engage in treatment, and it excludes those experiencing imminent safety risks, active psychosis, or limited cognitive abilities. It should be noted that families who find our services often have the resources to have already sought treatment that led to an OCD diagnosis and initial treatment engagement.

### 5.3. Conclusions

The review of our clinical data over the past three years provides guidelines for when and why patients might want to pursue specialty residential treatment. Families seeking support from our program often do so when functional impairment becomes too disruptive to everyday life, including family, social, and school relationships. Often, multiple attempts at therapy and medication trials have been made with limited success. The clinical profile of those engaging in our program presents with severe OCD, often involving various overlapping themes of OCD, combined with multiple comorbid psychiatric disorders, and a significantly impaired quality of life. Our program delivers evidence-based exposure treatment provided by an expert multi-disciplinary team that allows for individually tailored treatments. Targeting OCD in the milieu community setting allows for OCD-specific work to occur in real-time when the rituals are present, particularly rituals that dominate morning, evening, and meal routines. The milieu community also offers unique opportunities to connect with peers facing similar challenges.

As a residential program that offers evidence-based OCD treatment, we are uniquely suited to support our patients and families who are experiencing such incredible challenges in the face of debilitating OCD. Unfortunately, misunderstandings about our level of care and how our program works can contribute to families delaying seeking our services or are even discouraged from considering a higher level of care, falsely equating it to an inpatient service. For families that do find their way into our care, they more often than not are plagued by insurance challenges, either due to not having benefits that cover residential services, insurance companies holding our services to inpatient requirements (e.g., imminent safety risks), or limited time allowed in our treatment program leading families to make difficult decisions around terminating treatment early or paying out of pocket expenses. We have the opportunity to do incredibly meaningful work with our patients and families, yet more remains to be done to better understand pediatric OCD, refine treatments, and offer these evidence-based services in a way that is cost-effective and accessible to all in need.

## Figures and Tables

**Table 1:** Patient demographics.

Demographics	Total Sample	CARE Sample
*n*	168	120
Biological Sex		
%Female	71.2	63.2
%Male	27.6	36.8
%Other	1.2	N/a
Race/Ethnicity		
%White/Non-Hispanic	81.9	77.9
%Latino/Hispanic	4.8	8.0
%Asian	3.6	7.6
%Mixed Race	2.4	-
%Black/African American	1.8	0.8
%Native Hawaiian/Pacific Islander	0.6	0.8
%American Indian or Alaskan Native		1.5
%Other	1.8	4.6
Primary Language Spoken at Home		
%English	92	-
%English and a Second Language	6.6	-
%Non-English Language	1.2	-
Age		
*M* (*SD*)	*15.23 (1.68)*	*15.30 (2.11)*

**Table 2:** Psychiatric Medications at Time of Referral.

Medication Characteristics at Time of Referral	Active Medications	Previous Medications
Total patients (*n*)	166	
%Any SSRI, SNRI, or Clomipramine	84.9	57.8
*%SSRI*	75.9	57.2
*%SNRI*	6.6	10.2
*%Clomipramine*	4.2	1.8
%Other Antidepressants	13.3	9.0
*%Bupropion*	6.6	5.4
*%Trazodone*	3.6	1.2
*%Mirtazapine*	3.0	2.4
%SGAs	37.3	21.1
*%Risperidone*	10.2	5.4
*%Aripiprazole*	9.0	13.9
*%Quetiapine*	8.4	3.6
*%Olanzapine*	5.4	3.0
*%Lurasidone*	5.4	3.0
*%Cariprazine or Ziprasidone*	1.8	0
%Other OCD Augmenting Agents	4.8	0.6
*%N-Acetylcysteine*	4.2	0.6
*%Memantine*	1.2	0
%Benzodiazepines	9.0	3.6
%Other Anxiolytics or Sleep Aids	23.5	12.0
*%Hydroxyzine*	9.6	7.2
*%Melatonin*	6.0	0
*%Propranolol*	5.4	0.6
*%Clonidine*	4.2	1.8
*%Buspirone*	3.0	1.8
%ADHD Medications	22.3	19.3
*%Stimulant*	16.9	17.5
*%Guanfacine or Atomoxetine*	6.6	4.2
%Mood Stabilizers ^a^	10.2	2.4
Non-Psychiatric Medications or Supplements	32.5	—
*%Supplements*	13.9	—
*%Oral Contraceptives*	6.0	—
*%NSAIDs*	4.2	—
*%Oral Antibiotics*	3.6	—
*%Metformin*	2.4	—
*%Oral Immunosuppressants and Steroids*	1.2	—

^a^ Lamotrigine, lithium, valproic acid, gabapentin, or topiramate.

## Data Availability

The datasets presented in this article are not readily available because the data are part of an ongoing study. Requests to access the datasets should be directed to the corresponding author.
